# Minimal detectable difference of the finger and wrist range of motion: comparison of goniometry and 3D motion analysis

**DOI:** 10.1186/s13018-019-1177-y

**Published:** 2019-06-10

**Authors:** Lisa Reissner, Gabriella Fischer, Renate List, William R. Taylor, Pietro Giovanoli, Maurizio Calcagni

**Affiliations:** 1 Division of Plastic Surgery and Hand Surgery, University Hospital Zurich, University of Zurich, Raemistrasse 100, 8091 Zurich, Switzerland; 2 Institute for Biomechanics, ETH Zurich, Zurich, Switzerland; 3 Human Performance Lab, Schulthess Clinic, Zurich, Switzerland

**Keywords:** Manual goniometer, 3D motion capture, Range of motion, Motion analysis, Minimal detectable difference

## Abstract

**Background:**

The measurement of finger and wrist range of motion (ROM) is of great importance to clinicians when assessing functional outcomes of therapeutic interventions and surgical procedures. The purpose of the study was to assess the repeatability of ROM measurements of the hand joints with manual goniometer and 3D motion capture system and to calculate the minimal detectable difference for both methods.

**Methods:**

Active finger and wrist joints ROM of 20 healthy volunteers were assessed using a manual goniometer and 3D motion capture system. Minimal detectable difference (MDD) and standard error of measurement (SEM) were calculated for both measurement systems and compared within the same task. Maximal ROM of all joints was registered twice on two different days to evaluate the test-retest repeatability. The intraclass correlation coefficients (ICC) was calculated and examined to determine if reliability ≥ 0.70 existed.

**Results:**

MDD for the 3D motion capture was between 5 and 12° except for the metacarpophalangeal joint (MCP) 1, interphalangeal joint (IP), and MCP5. SEM values lay between 2 and 4° for all joints except for the MCP5, IP, and MCP1. For the goniometric measurements, MDD and SEM were between 12–30° and 4–11°, respectively. The reliability criterion (ICC > 0.7) was achieved for the ROM measurement with the 3D motion capture system for 94% of the joints and in only 65% of the joints with the manual goniometer.

**Conclusions:**

Joint ROM assessed with 3D motion analysis showed higher test-retest agreement demonstrating overall better repeatability for this method. Because of the smaller measurement error, the 3D motion capture system has a smaller MDD. Only individual test-rest differences bigger than the MDD can be considered as real changes, and therefore, in an experimental situation, the use of a more precise measurement method can greatly reduce the number of subjects needed for a statistical significance. Goniometer measurements of some joints should be carefully interpreted, due to a low repeatability and reliability.

**Trial registration:**

This study is approved by the Ethical Committee Zurich (Kek-ZH-Nr: 2015-0395).

## Introduction

The measurement of finger and wrist postures is one of the important parameters for the clinicians when assessing the outcomes of therapeutic interventions and compare them. Joint angular measurements are also essential for hand therapists to record the progress of rehabilitation. It is therefore important for clinicians and researchers to have complete and relevant information on the accuracy, repeatability, and reliability of these measurements. While the manual goniometer is commonly used in clinical practice as a tool to measure joint angles, 3D motion capture systems are increasingly applied in research to measure hand motion [1–5]. Moreover, 3D motion capture systems allow the dynamic evaluation of all hand joints simultaneously [6–8]. They determine the position of skin markers highly accurate. The main advantages of the manual goniometry are that it is cheap, fast, and does not require data post-processing or joint angle calculations, and the main drawback is that it relies on rater’s performance for the quality of measurements. For evaluative instruments that are used to measure changes in the same subject over time, the ability to detect minimal clinically important differences is essential. Hence, it is fundamental to know the size of the measurement error is required not only for the selection of the appropriate measurement tool, but also for the interpretation of the data and the comparison between different studies.

Trained therapists generally have adequate intrarater reliability for the measurement of wrist and finger postures; however, some joints are easier to assess [9–12]. When standard goniometry is used, variability between 2 and 7° occurs in joint angle measurements of the hand [13–15]. The validation of goniometer measurement was done in splinted positions, which means that the force applied on the joint by the examiner was neutralized. This is not the case in real life where joints are examined looking for the actual angles and not a predefined one [16, 17]. Moreover, there are few studies comparing manual goniometer measurement and 3D motion capture, but none of them taking into account all the joints of the hand [16].

Sample size calculation (power) is a standard requirement for high-quality studies. Minimal detectable difference (MDD) and standard error of measurement (SEM) are among the most important parameters for its calculation. If we can reduce them, this will result in a smaller sample size.

Therefore, the aim of this study was to assess the repeatability of ROM of the hand joints with manual goniometer and 3D motion capture system and to calculate the minimal detectable difference and the standard error of measurement for both methods.

## Material and methods

### Participants and protocol

Finger and wrist joints motion of 20 healthy right-hand dominant volunteers (ten men, ten women) with mean age 28 years (SD 4.7) were assessed with 3D motion capture system and measured with manual goniometer. In order to assess the test-retest repeatability for both methods, each participant was tested on two different days. The same hand surgeon performed all goniometric measurements, and the same examiner placed the skin markers in both sessions. The local ethics committee approved the study, and all participants provided written informed consent for their data to be used for this analysis (Kek-ZH-Nr: 2015-0395).

### Manual goniometric measurements

The protocol of the goniometric measurement followed the recommendations of the American Society for Hand Therapists for the wrist joint and the finger joints [17–19]. Each volunteer placed their elbow on the table with the forearm in neutral position. Dorsal placement of a plastic goniometer (Zimmer®) on the wrist was applied. For the measurement of the distal interphalangeal joint (DIP), raters instructed patients to maximal extend the metacarpophalangeal joint (MCP) while maximal flexion of the proximal interphalangeal (PIP) joint and DIP joint, making a hook fist. During pronation and supination of the forearm, the volunteer was sitting with the shoulder in 0° of flexion, extension, abduction, and rotation so that the upper arm was close to the side of the body. The elbow was in 90° of flexion, and the goniometer was placed just proximal to the radial and ulnar styloid process while performing maximal pronation and supination.

### 3D motion capture system and setup

**Fig. 1:**
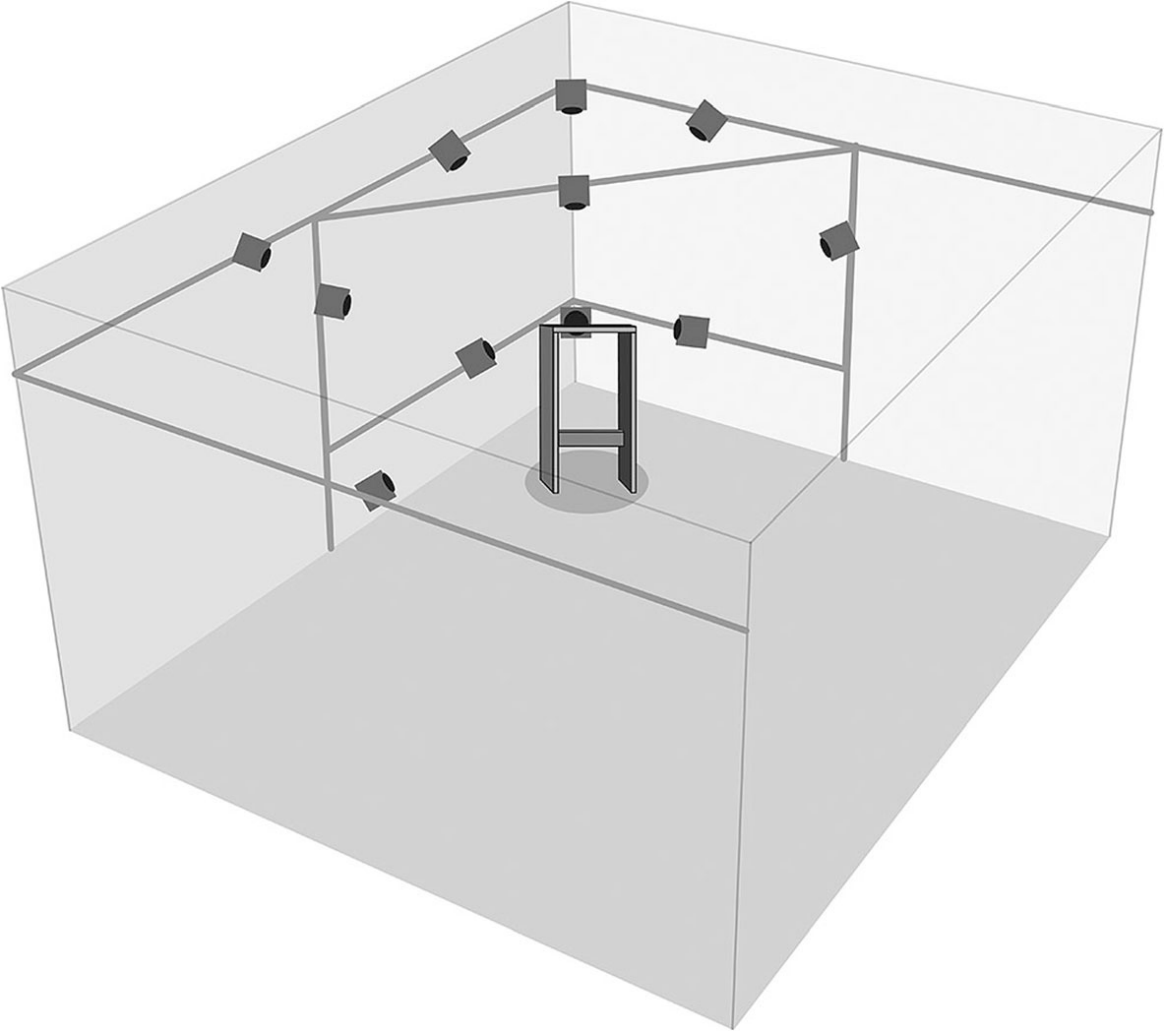
Camera setup

An optoelectronic motion capture system consisting of 11 fixated infrared cameras (VICON® MX3+ and VICON® MX3 motion capture system, Oxford Metrics Ltd., UK) and the corresponding software VICON®-Nexus (version 2.3) were used for data collection. The capture volume was approximately 50 × 50 × 50 cm^3^, and the cameras were positioned such that the markers were always visible by at least two cameras, avoiding hiding of markers (Fig. 1). The cameras have a resolution of 659 × 493 pixel, and recordings were carried out with a frequency of 100 Hz.

**Fig. 2:**
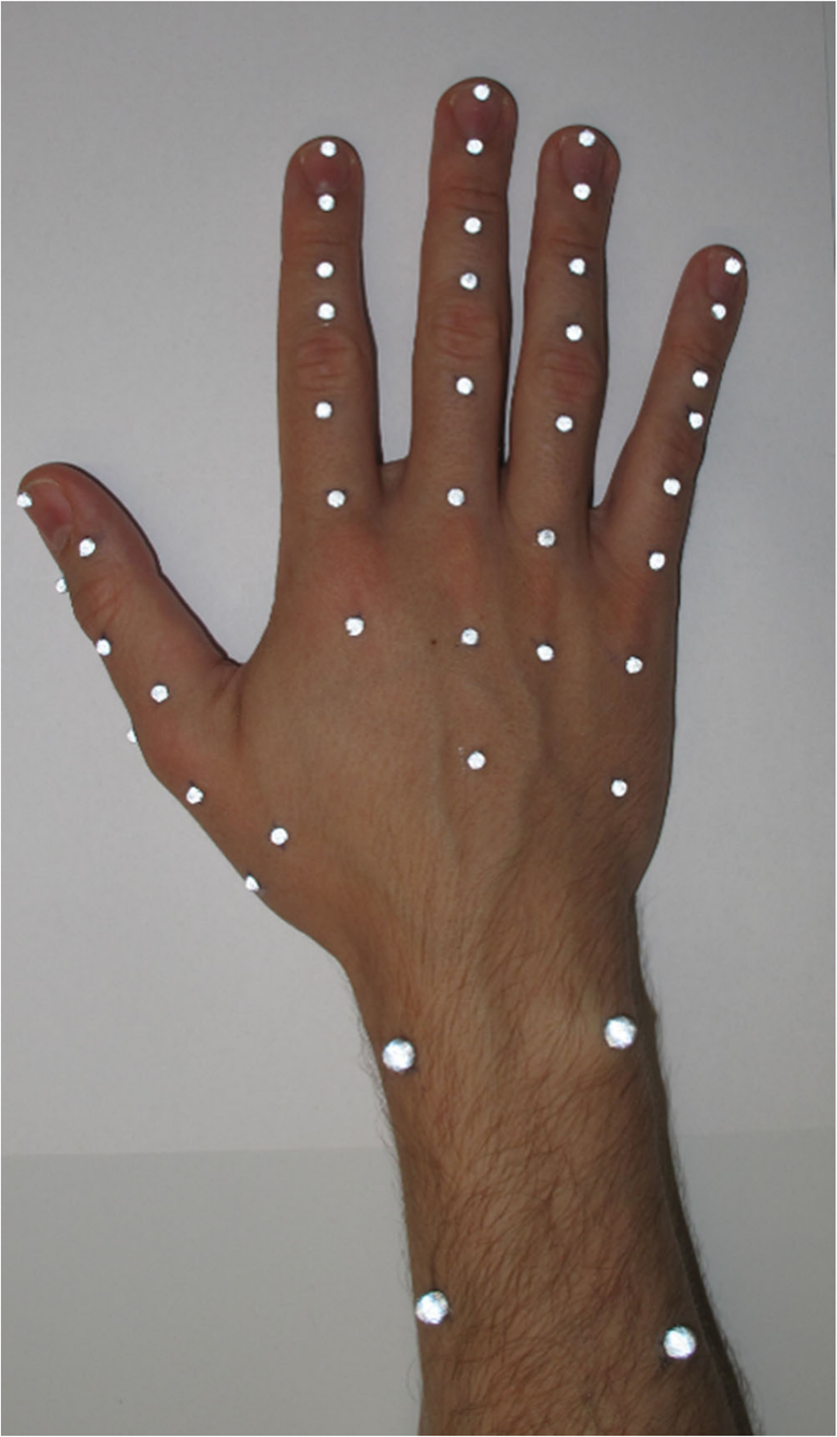
Marker position of the fingers and the wrist

In total, 46 reflective markers were placed at specific positions on the finger, wrist, and forearm (Fig. 2). The three markers on the elbow are located on the lateral and medial epicondyle as well as proximal of the olecranon. The spherical markers at the elbow and forearm had a diameter of 9 mm and 5 mm, respectively. For the hand and fingers, hemispherical markers with a diameter of 3 mm were chosen. We attached the markers with a skin-compatible adhesive tape.

### Motion tasks

First, a natural reference position with the hand lying on a flat surface and a 40° wedge between the second finger and the thumb was recorded. Afterwards, each volunteer performed a set of basic motion tasks:

- Pronation-supination (P/S) of the forearm
- Flexion-extension (F/E) and radial-ulnar deviation (R/U) and of the wrist
- Combined F/E in the MCP, PIP, and DIP joints of the long fingers (make a fist)
- F/E of the PIP and DIP joints of the long fingers (without movement in the MCP joints)
- F/E of the thumb

The tasks aimed to detect joint ROM in a single movement plane. Furthermore, they were used to calculate functional joint axes and centers. Each trial started and ended with the hand in a neutral position and consisted of three cycles (e.g., flexion-extension-flexion-extension-flexion-extension). Five valid repetitions of each task were recorded per session.

### Data processing and data analysis

For the kinematic description of the hand, 18 segments were defined and considered as rigid bodies. At least three markers per segment are needed to allow kinematic analysis in all three movement planes. The kinematic model of the fingers was based on the assumption that only motion around the flexion axis is possible for the PIP and DIP joints. Therefore, kinematics of the long fingers could be assessed with a reduced marker concept using only two longitudinally aligned markers per segment. The F/E angles were calculated by means of the vectors between the markers of each finger segment similar to Metcalf et al. but with a marker proximal or distal of the joint defining the segment vector instead of markers on the joint [20, 21].

The kinematic evaluation of the distal radioulnar joint, the wrist joint, and the thumb joints was based on marker clusters. As described in List et al., the rotation of a segment relative to a static reference position was estimated during the dynamic trials using a least squares fit [22]. Then, joint kinematics was expressed as the relative rotation matrix of the distal segment relative to the proximal segment. To determine the joint centers and joint axes from specific calibration movements, a functional approach from List et al. was adapted to the thumb and wrist [22]. Joint rotations were calculated according to Grood and Suntay and in accordance with the standards defined by the International Society of Biomechanics [23, 24].

After checking recorded data for errors, the data were summarized using descriptive statistics. All analyzed parameters and their abbreviations are defined in Table 1. For all abbreviations of these parameters, a subscripted character G refers to the goniometer measurement and subscripted character V refers to the analysis by means of the motion capture system.

**Table 1:** Kinematic parameters

Parameter	Abbreviation	Description/formula
Measurement parameter-determined for each subject in measurement session 1 + 2 (test-retest)		
Range of motion	ROM_G1/G2,_ ROM_V1/V2_	Maximum range of motion measured within a method and session
Test-retest difference	DIFF_G_/DIFF_V_	Individual test-retest difference of ROM: DIFF_G_ = ROM_G1_ − ROM_G2_/DIFF_V_ = ROM_V1_ − ROM_V2_
Test-retest and repeatability parameter—between test and retest within the same method over all subjects		
Mean difference	meanD_G_/meanD_V_	Mean DIFF_G_/DIFF_V_
Standard deviation of the difference	SDD_G_/SDD_V_	Standard deviation of DIFF_G_/DIFF_V_
Standard error of measurement	SEM_G_/SEM_V_	Estimated measurement precision according to de Vet^24^: SEM = SDD/√2
Minimal detectable difference	MDD_G_/MDD_V_	MDD = 1.96 × SDD
% within 5° or 10°	PD_G_ < 5° or < 10°/PD_V_ < 5° or < 10°	Percentage of subjects with an absolute |DIFF_G_|/|DIFF_V_| smaller than 5° or 10°
Intraclass correlation coefficient	ICC_G_/ICC_V_	Intraclass correlation coefficient (3,1) of the test-retest measurement of the ROM
Method comparison—between measurement methods		
Mean ROM	Mean_romG_/Mean_romV_	Mean ROM of test and retest of each subjects averaged over all subjects
Standard deviation of ROM	SD_romG_/SD_romV_	Standard deviation of Mean_romG_/Mean_romV_

For the dynamic trials recorded by the motion capture system, the minimum and maximum joint angle (e.g., maximum flexion and extension position) was determined for each trial and averaged within the five trials of the same session to obtain the ROM. The individual test-retest difference (DIFF) of the ROM was determined for each subject. To quantify the test-retest repeatability of the ROM, SEM and intra-class correlation coefficient (ICC) were calculated within the same task and method for all joints. It is recommended that ICC values need to be greater than or equal to 0.70 to be considered acceptable as a clinically meaningful measurement tool [25]. According to de Vet et al., the SEM represents measurement error and equals the square root of the variance of differences [26]. Only changes of the ROM that exceed the variability induced by the method can be regarded as real changes [27]. Therefore, based on the SEM, the minimal detectable difference (MDD) of both methods was calculated. In addition, the percentage of subjects with absolute test-retest differences below 5° and 10° (PD < 5°/PD < 10°) were determined for both methods, respectively.

## Statistical analysis

Two-tailed *t* tests (*p* ≤ 0.05) were performed to compare motion analysis and goniometry regarding the mean test-retest difference (meanD_V_ vs. meanD_G_) as well as the average ROM (Mean_romV_ vs. Mean_romG_). For a healthy population, we assumed ROM to be constant over time. Therefore, DIFF_G_ and DIFF_V_ are considered as an estimation of measurement error, and a limit of 5° was set based upon the accuracy of the manual goniometer shown to be around 5° in literature [28]. The null hypotheses were that:

(H_01_) Within the same method, meanD equals zero, (H_02_) DIFF lies within 5°, and (H_03_) the ROM is equivalent when measured with the goniometer or the motion capture system.

H_01_ was rejected, if the absolute value of the ratio of meanD and standard deviation of difference (SDD) exceeds the 5% level of agreement (|T| > 1.96; *p* ≤ 0.05). H_02_ was rejected, if the estimated precision represented by the SEM was > 5°.

## Results

For the 3D motion capture system, no valid joint angle could be calculated for four subjects at the radioulnar joint and for one subject at the MCP5 joint due to issues with visibility of markers or a lost marker at the elbow. Furthermore, one subject had a misplaced marker on the thumb, affecting MCP1 calculations, and one subject had a shifted marker affecting MCP4 and MCP5 angle calculations. Therefore, these values had to be excluded from further data analysis after visual inspection of the recorded data. The available data are reported in the second column of Table 2.

**Table 2:** Test-retest results of goniometer and 3D motion analysis measurement

Joint	Direction	Motion analysis						Goniometer					
		*n*_V_	MDD_V_ [°]	SEM_V_ [°]	ICC_V_	PD_V_ < 5° [%]	PD_V_ < 10° [%]	*n*_G_	MDD_G_ [°]	SEM_G_ [°]	ICC_G_	PD_G_ < 5° [%]	PD_G_ < 10° [%]
Radioulnar	P/S	16	8	3.0	0.94	69	100	20	19	6.8	0.35	45	65
Wrist	F/E	20	6	2.1	0.97	95	100	20	18	6.4	0.87	50	75
Wrist	R/U	20	7	2.6	0.90	90	95	20	18	6.5	0.72	55	65
MCP2	F/E	20	7	2.7	0.96	80	100	20	19	7.0	0.79	25	60
MCP3	F/E	20	9	3.1	0.95	65	100	20	24	8.8	0.59	35	60
MCP4	F/E	19	9	3.4	0.92	47	100	20	18	6.6	0.81	40	60
MCP5	F/E	18	16	5.7	0.90	33	72	20	22	8.0	0.86	20	40
PIP2	F/E	20	8	3.0	0.85	85	95	20	12	4.2	0.66	55	80
PIP3	F/E	20	12	4.2	0.66	65	85	20	12	4.3	0.64	55	80
PIP4	F/E	20	10	3.8	0.86	80	85	20	13	4.6	0.60	60	75
PIP5	F/E	20	11	4.0	0.85	65	90	20	15	5.5	0.40	45	65
DIP2	F/E	20	9	3.4	0.92	65	90	20	14	5.0	0.79	45	80
DIP3	F/E	20	9	3.3	0.92	60	100	20	18	6.7	0.75	30	60
DIP4	F/E	20	10	3.5	0.96	70	95	20	17	6.3	0.84	40	65
DIP5	F/E	19	11	4.0	0.94	58	95	20	14	4.9	0.86	60	85
IP	F/E	20	14	5.0	0.93	50	80	20	30	11.0	0.85	35	50
MCP1	F/E	19	14	5.0	0.91	68	79	20	19	6.8	0.83	30	70

There was a wide range of maximum ROM among the healthy subjects (SD_romV_ 9°, SD_romG_ 10°) (Tables 3 and 4). The two-tailed *t* test revealed significant (*p* < 0.05) differences of the Mean_rom_ derived from the different methods only for the pronation-supination movement of the radioulnar joint. For this joint, Mean_romG_ and Mean_romV_ differ by almost 57°, with the lower values measured with the 3D motion capture system.

**Table 3:** Range of motion (mean and SD) for goniometer and 3D motion analysis measurement

Joint	Direction	Measurement day 1					Measurement day 2				
		Motion analysis		Goniometer			Motion analysis		Goniometer		*P* value
		Mean_romV1_ [°]	SD_romV1_ [°]	Mean_romG1_ [°]	SD_romG1_ [°]	*P* value	Mean_romV2_ [°]	SD_romV2_ [°]	Mean_romG2_ [°]	SD_romG2_ [°]	
Radio-ulnar	P/S	116	10	174	8	< 0.0001	114	10	170	8	< 0.0001
Wrist	F/E	150	9	141	14	0.570	150	10	139	12	0.499
Wrist	R/U	51	7	56	10	0.663	51	6	58	10	0.573
MCP2	F/E	109	10	114	12	0.787	109	9	116	13	0.663
MCP3	F/E	114	10	114	11	0.991	113	10	115	13	0.902
MCP4	F/E	113	11	115	10	0.883	110	10	119	14	0.644
MCP5	F/E	115	16	120	14	0.795	112	13	123	19	0.624
PIP2	F/E	118	6	109	6	0.275	118	6	113	7	0.617
PIP3	F/E	119	6	109	6	0.234	118	6	112	7	0.487
PIP4	F/E	122	7	109	6	0.186	121	8	112	7	0.411
PIP5	F/E	105	7	101	5	0.705	106	9	103	8	0.775
DIP2	F/E	80	10	84	9	0.751	83	10	84	9	0.899
DIP3	F/E	93	9	95	12	0.927	95	9	94	9	0.921
DIP4	F/E	84	13	87	12	0.882	85	13	89	12	0.833
DIP5	F/E	89	13	89	10	0.996	88	12	90	11	0.906
IP	F/E	93	14	103	23	0.720	92	13	104	21	0.643
MCP1	F/E	72	15	71	14	0.947	70	12	72	11	0.964

Significant difference between motion capture and goniometer measurement

**Table 4:** Range of motion (median and range) for goniometer and 3D motion analysis measurement

Joint	Direction	Measurement day 1						Measurement day 2					
		Motion analysis			Goniometer			Motion analysis			Goniometer		
		Median romV1 [°]	Min romV1 [°]	Max romV1 [°]	Median romG1 [°]	Min romG1 [°]	Max romG1 [°]	Median romV2 [°]	Min romV2 [°]	Max romV2 [°]	Median romG2 [°]	Min romG2 [°]	Max romG2 [°]
Radio-ulnar	P/S	117	101	136	172	160	190	114	99	137	170	156	182
wrist	F/E	151	129	166	141	102	168	150	127	165	139	105	160
wrist	R/U	52	37	62	57	42	82	52	40	62	58	44	84
MCP2	F/E	108	91	132	112	90	138	108	94	127	116	96	136
MCP3	F/E	112	98	137	115	98	134	112	99	133	115	96	138
MCP4	F/E	112	100	138	115	96	134	107	93	130	119	100	148
MCP5	F/E	116	90	151	121	98	151	110	93	137	123	94	162
PIP2	F/E	118	110	132	108	98	118	119	109	128	113	102	132
PIP3	F/E	120	109	129	108	98	118	118	108	131	112	102	126
PIP4	F/E	123	110	135	108	100	120	122	105	133	112	100	122
PIP5	F/E	105	90	120	102	84	110	106	83	119	103	90	114
DIP2	F/E	79	65	98	84	66	104	84	66	100	84	62	96
DIP3	F/E	92	77	107	94	70	114	98	81	109	94	76	108
DIP4	F/E	87	58	103	89	68	114	85	61	105	89	64	104
DIP5	F/E	93	61	107	91	74	108	88	70	108	90	70	110
IP	F/E	93	67	114	101	66	152	92	58	111	104	70	160
MCP1	F/E	72	47	110	70	50	104	70	50	97	72	56	98

The results of the test-retest parameters are presented in Table 2. No statistically significant difference (H_01_) of the ROM (*p* level ≤ 0.05) was found between the first and second measurement for both methods in all joints.

For the 3D motion capture method, MDD_V_ lay between 5 and 12° except for the MCP1, IP, and MCP5. For the goniometric measurements, MDD_G_ was between 12 and 30°.

**Fig. 3:**
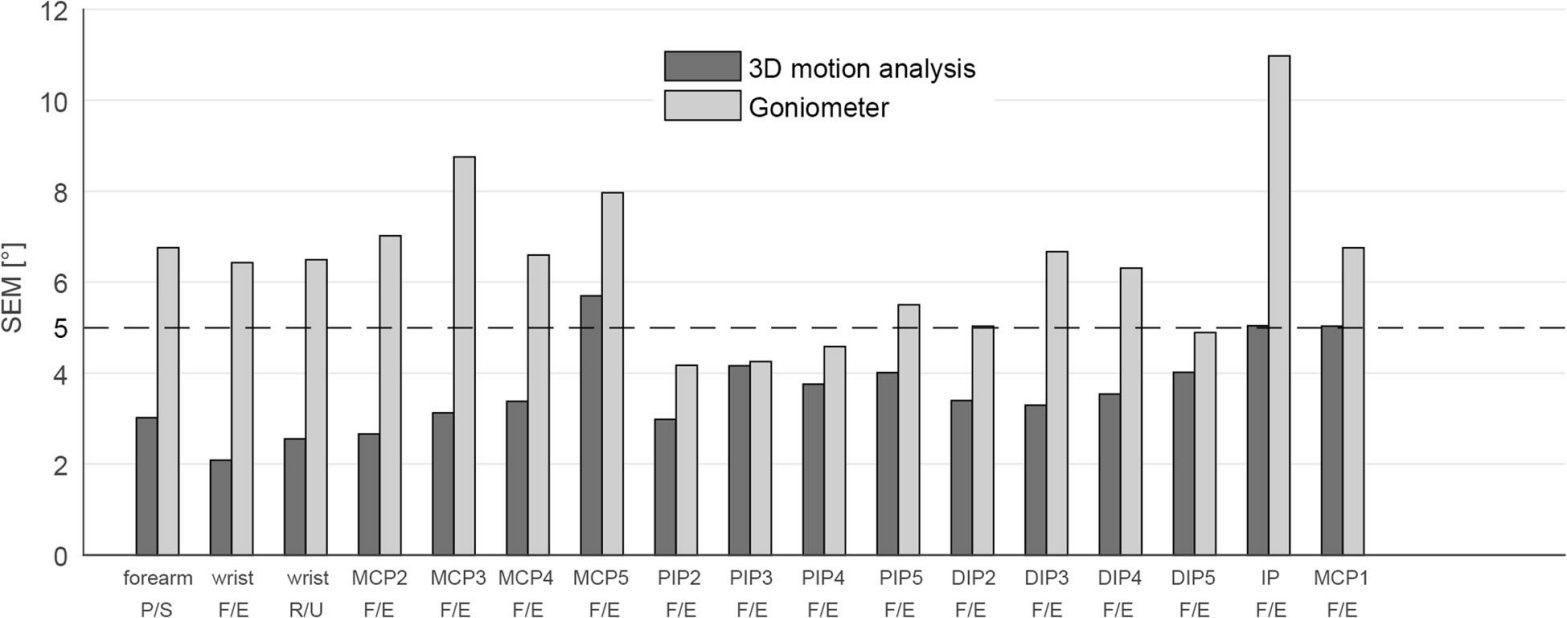
SEM of 3D motion analysis and goniometer of all joints

The observed precision of the measurement represented by the SEM is displayed in Fig. 3 and Table 2. SEM_V_ lied below the limit of 5° for all joints except for the MCP5 (SEM_V_ 5.7°), IP (SEM_V_ 5.0°), and MCP1 (SEM_V_ 5.0°). For the goniometric measurements, SEM_G_ exceeds the limit of 5° (SEM_G_ 5.0–11.0°) in all joints, except for PIP2-4 and DIP5 (SEM_G_ 4.2–4.9°).

ICC ranged from 0.35 to 0.87 (ICC_G_) and 0.66 to 0.97 (ICC_V_) for the goniometric and 3D motion capture measurements, respectively (Table 2). Six out of 17 joints (radioulnar, MCP3, PIP2-5) did not achieve the reliability criterion with the manual goniometer. In comparison, the ICC_V_ value for the 3D motion capture system was higher for all degrees of freedom, and the ROM measurements with the motion capture system met the reliability criterion for all joints except for the PIP3.

Overall, 43% and 67% of the goniometric measurements had a test-retest difference below 5° (PD_G_ < 5°) and 10° (PD_G_ < 10°), respectively (Table 2). The corresponding percentages for the 3D motion capture system were 67% (PD_V_ < 5°) and 92% (PD_V_ < 10°), respectively. The DIP5 was the only joint for which slightly more volunteers had an angular difference of less than 5° for the goniometric measurements compared to the 3D motion capture system. For all other joints, the 3D motion capture system had a higher percentage of individuals with small (< 5°) inter-session differences.

## Discussion

In this study, we assessed the repeatability of ROM measurements of the hand joints with 3D motion capture system, compared them with manual goniometry, and calculated the MDD for both methods. We measured all joints of the fingers and the wrist.

We observed method differences from − 57 to + 11°, where negative values indicate higher ROM when examined with the goniometer (Mean_romG_ > Mean_romV_).

Since the true value of the joint angle is unknown, the comparison between the two methods serves as the first step in the validation of the new motion analysis protocol. Skin movement relative to the bone is the biggest source of error in motion analysis with skin markers [29, 30]. Longitudinal rotations are more affected from skin movement artifacts, therefore leading to an under-/overestimation of the joint angle in motion analysis [30, 31]. In agreement, we found the highest method disagreement (57°) and the only significant difference for the pronation-supination movement. Difficulties to measure the radioulnar joint with a goniometer (ICC 0.35_G_, SEM_G_ 6.8°) might have further contributed to the large difference between the methods.

Armstrong et al. suggest that the lack of precision in goniometric measurement could be technique related, as the current method of measuring true forearm rotation involves placing a flat goniometer along the curved surface of the flexion/extension crease of the wrist [32]. The observed significant difference between the methods for measuring forearm rotation indicates that adjustments to the methodology are necessary. Schmidt et al. propose a procedure to reduce the influence of skin movement artifacts by looking at the hand rotation during pronation-supination instead of the rotation of the forearm itself [33]. Implementation of such simple corrections might further improve the presented measurement method.

A comparison with a gold standard, such as an imaging technique, would be a possibility to estimate the accuracy of the measurements. However, the validity of the data is beyond the scope of this article. Still, our results reveal that the measurement system used to obtain the ROM has to be considered for clinical data interpretation. Therefore, a specific norm database for every method is highly recommended.

n a clinical setting, methods are often used to evaluate the effects of interventions or monitor changes over time within the same subject. Therefore, a focus on agreement parameters is recommended by de Vet et al. [26]. The SEM and MDD express measurement error in the same unit as the original value, which facilitates clinical interpretation. In contrast to ICC, SEM and MDD are not influenced by variability among the sample [34]. Hence, their values can be transferred to various groups of patients.

Averaged over all analyzed joints, the MDD_V_ was 10°, compared to 18° MDD_G_. Therefore, measurements by means of a motion capture system allow us to recognize smaller changes in joint mobility than with goniometer. This means that we have to be very careful in the clinical setting to interpret a change in the ROM as a true change, or just as a measurement error. For the wrist joint flexion/extension and radial/ulnar deviation, MDD_G_ was 18°. Macedo and Magee examined the passive ROM of the wrist with a universal goniometer in 12 healthy adults. They found a MDD_G_ for the wrist flexion of 11° and for the wrist extension of 8°, which is lower than our MDD_G_, but higher than our MDD_V_ [35].

The MDD_G_ for the finger joint lied between 12° and 24°. Ellis and Bruton examined the finger joints with a goniometer, but in a splinted position, so they had MDD (reported as 95% confidence interval of difference) of between 4° and 5° [1].

Overall, the calculated precision of the ROM measurements was SEM_V_ 3.6° and SEM_G_ 6.4° for the 3D motion capture system and goniometer, respectively. The mean values of all repeatability parameters indicate higher test-retest agreement for the 3D motion capture method. For the wrist joint, the SEM_G_ with the goniometer was 6.4° (F/E) and 6.5° (R/U). LaStayo and Wheeler assessed the passive ROM of the wrist with a universal goniometer in 120 patients with wrist conditions. They reported SEM between 5.6° and 8.1°, like Macedo and Magee with SEM between 2.9° and 7.4°, compare to Horger who calculated a SEM between 2.6° and 4.5° [12, 35, 36]. Our results consider the ROM of the movement, so both measurement points (e.g., maximum flexion and extension position) are affected from independent error associated with the placement of the goniometer, whereas the other studies showed the results of each direction separately.

For the finger joints, SEM_G_ was between 4.2° and 8.8°. Stam et al. evaluated 20 healthy subjects with a goniometer while holding cylinders with different diameter and had a SEM between 4° and 6°, similar to our results [37].

In comparison with previous repeatability goniometry studies, the intrarater reliability for the active ROM of the middle finger found in our study lies within the range of the intra- and interrater reliability (ICC 0.43 to 0.99) determined by Lewis et al. [2]. Solgaard et al. assessed intraobserver SDD for the goniometry of the wrist of 5.2–8° [38]. Compared to these findings, our results for wrist goniometry are slightly higher. In contrast, the 3D motion capture of the wrist ROM had better repeatability than the goniometry results in both studies.

Compared to previous measurements by 3D motion capture, we found excellent test-retest reliability on the wrist (ICC 0.90–0.97). The corresponding values in Levanon et al. were only good (ICC 0.77–0.83) [39]. In contrast, the root-mean-square error in our study was 5.3°, whereas Sancho-Bru et al. found smaller errors in repeatability (3.4°) [8]. In that study, a different marker set was used and the repeatability was assessed for grasping different objects while we analyzed the maximum ROM. It is possible that variability of the ROM movement is bigger than in specific grasping tasks, but to quantify the source of error, a validation would be needed.

### Limitations and achievements

In this study, we do not simply compare two different measurement tools but rather two different measurement procedures. Therefore, the comparison includes methodological differences in addition to the measurement system itself, which might have influenced the reported repeatability.

In manual goniometry, every joint was assessed separately. The kinematic analysis resulted in a continuous angular curve, from which the ROM was extracted. In comparison, the goniometer had to be placed twice for each joint in order to obtain the ROM, always in an interaction with the subject, which can influence the result. Hence, both measurement points (e.g., maximum flexion and extension position) are affected from independent error associated with the placement of the goniometer, whereas the position of the skin markers stayed the same during the whole session (compensation of error possible). We do not see this as a limitation of the study, but rather as an advantage of the motion analysis method.

The main difference between the two protocols was the amount of measurements of each joint angle. When using the motion capture system, the maximum ROM of the dynamic trials could be averaged within the session, which might have compensated for outliers. In contrast, with the manual goniometer, each parameter was measured only once per session, as otherwise the rater could recall the values. This difference has likely contributed to the better results for the 3D motion capture system. Still, the study implements both methods such as they are usually applied in a clinical setting. It quantifies the test-retest repeatability of a realistic application, where usually a single surgeon or therapist measures the ROM to monitor change during treatment. We are aware that we cannot make a statement about the accuracy of both methods, the 3D motion capture system and the goniometry. A comparison with a radiological examination would be necessary for this. As a first step, we concentrated on the repeatability of both methods and on their comparison, since repeated tests to assess changes are very common in the clinical setting and the research [26].

We missed few measurements because of marker loss. This could happen while they are attached only with a double-sided adhesive tape, and can get lost or displaced, which is a disadvantage in motion analysis. The advantage of the 3D motion capture system is the dynamic evaluation of the wrist and all finger joints simultaneously. Therefore, it can be applied for the assessment of the ROM as well as dynamic functional tasks, such as activities of daily living. The main advantages of the manual goniometry are that it is much easier to implement in the clinical setting. Our study shows that in applications where the goniometer is not precise enough, motion analysis is a possible alternative due to its lower MDD. The choice of the method has to be in accordance with the research question and the expected or clinically relevant change in joint ROM.

## Conclusion

In conclusion, the MDD of the 3D motion capture system is smaller than of the goniometer measurement. This is particularly important in an experimental setup where a higher degree of precision is requested. In the clinical research, better MDD permits relevant reduction of the sample size.

## Abbreviations

DIFF: Test-retest difference
DIP: Distal interphalangeal joint
ICC: Intraclass correlation coefficient
IP: Interphalangeal joint
MCP: Metacarpophalangeal joint
MDD: Minimal detectable difference
PIP: Proximal interphalangeal joint
ROM: Range of motion
SEM: Standard error of measurement
